# In Vitro Activity of Pentamidine Alone and in Combination with Antibiotics against Multidrug-Resistant Clinical *Pseudomonas aeruginosa* Strains

**DOI:** 10.3390/antibiotics9120885

**Published:** 2020-12-09

**Authors:** Soraya Herrera-Espejo, Tania Cebrero-Cangueiro, Gema Labrador-Herrera, Jerónimo Pachón, María Eugenia Pachón-Ibáñez, Rocío Álvarez-Marín

**Affiliations:** 1 Institute of Biomedicine of Seville (IBiS), Virgen del Rocio University Hospital/CSIC/University of Seville, 41013 Seville, Spain; 11.sorayaherrera.2b@gmail.com (S.H.-E.); tany5_91@hotmail.com (T.C.-C.); gemalabrador@hotmail.com (G.L.-H.); pachon@us.es (J.P.); rocioalma@gmail.com (R.Á.-M.); 2 Clinical Unit of Infectious Diseases, Microbiology, and Preventive Medicine, Virgen del Rocio University Hospital, 41013 Seville, Spain; 3 Department of Medicine, University of Seville, 41009 Seville, Spain

**Keywords:** *Pseudomonas aeruginosa*, multidrug-resistance, antibiotics, pentamidine, in vitro activity

## Abstract

Multidrug-resistant (MDR) *Pseudomonas aeruginosa* is a public health problem causing both community and hospital-acquired infections, and thus the development of new therapies for these infections is critical. The objective of this study was to analyze in vitro the activity of pentamidine as adjuvant in combinations to antibiotics against seven clinical *P. aeruginosa* strains. The Minimum Inhibitory Concentration (MIC) was determined following standard protocols, and the results were interpreted according to the European Committee on Antimicrobial Susceptibility Testing (EUCAST) breakpoints; however, the gentamicin activity was interpreted according to the Clinical and Laboratory Standards Institute (CLSI) recommendations. The bactericidal in vitro activity was studied at 1×MIC concentrations by time–kill curves, and also performed in three selected strains at 1/2×MIC of pentamidine. All studies were performed in triplicate. The pentamidine MIC range was 400–1600 μg/mL. Four of the strains were MDR, and the other three were resistant to two antibiotic families. The combinations of pentamidine at 1×MIC showed synergistic activity against all the tested strains, except for pentamidine plus colistin. Pentamidine plus imipenem and meropenem were the combinations that showed synergistic activity against the most strains. At 1/2×MIC, pentamidine plus antibiotics were synergistic with all three analyzed strains. In summary, pentamidine in combination with antibiotics showed in vitro synergy against multidrug-resistant *P. aeruginosa* clinical strains, which suggests its possible use as adjuvant to antibiotics for the therapy of infections from MDR *P. aeruginosa*.

## 1. Introduction

*Pseudomonas aeruginosa* is an opportunistic pathogen associated with an ever-widening array of life-threatening acute and chronic infections, including diverse respiratory-tract infections (ventilator-associated pneumonia, chronic infection of bronchiectasis, and cystic fibrosis), urinary tract infections, bacteremia, and central nervous infections [1]. Moreover, chronic infections caused by *P. aeruginosa* are a major worry as their spread and mortality continue to increase. These infections are majorly attributed to biofilm formation plays an essential role in the attachment of this bacteria on surfaces, and then contributing to multi-drug resistance in this pathogen [2].

P. aeruginosa is one of the pathogens included in the group “ESKAPE” (Enterococcus faecium, *Staphylococcus aureus*, *Klebsiella pneumoniae*, *Acinetobacter baumannii*, *P. aeruginosa*, and *Enterobacter* species), with a worldwide impact on hospital infections and an extensive ability to develop resistance to antibacterial drugs. Thus, *P. aeruginosa* is one of the pathogens included in the critical category of the World Health Organization (WHO)’s priority list of bacterial pathogens for which research and the development of new antibiotics is urgently required [3]. *P. aeruginosa* was reported by the European Centre for Disease Prevention and Control (ECDC) Epidemiological Report 2016 on healthcare-associated infections, to be the most frequently isolated microorganism in intensive care unit (ICU)-acquired pneumonia and one of the most frequently isolated in blood-stream infections and urinary tract infections [4].

The 2018 ECDC surveillance of antimicrobial resistance in Europe report [5] detailed that *P. aeruginosa* resistance to two or more antimicrobial groups was common and seen in 19.2% of all tested isolates. Also, they report large inter-country variations for all antimicrobial groups, with generally higher resistance percentages reported from Southern and Eastern Europe than Northern Europe. As an example, in 2018, the % of *P. aeruginosa* isolates with multidrug-resistance (MDR), resistance to three or more antimicrobial families among piperacillin/tazobactam, ceftazidime, fluoroquinolones, aminoglycosides, and carbapenems in Germany, Spain and Greece are 6.0%, 10.9%, and 28.7%, respectively. A recent study in India observed that the majority of hospital-acquired isolates were MDR, pointing out an 89.5% of analyzed *P. aeruginosa* isolates sensitive only to colistin [6]. Also, in a recent study in the United States about respiratory infections due to MDR P. aeruginosa, they showed that among the total laboratory-confirmed P. aeruginosa isolates from respiratory sources, 27.5% (523/1904) of them were MDR [7].

Finally, infections caused by multidrug-resistant (MDR) *P. aeruginosa* are associated with a higher morbidity and mortality, and the associated healthcare burden requires an urgent focus on infection prevention, adequate infection management, and the development of novel therapies to treat this infections [8,9].

As a new strategy, the repurposing of drugs for the treatment of infections by MDR *P. aeruginosa* has several advantages, such as reducing the time, cost, and risk associated with the development of new antimicrobials [10]. The repurpose of several drugs has been proven to have activity against bacterial infections. Ciclopirox, used for decades as antifungal agent, was reported to have direct antibacterial activity against several of the high-priority MDR Gram-negative bacteria [11,12]. The study by Ayerbe-Algaba et al. also showed that oxyclozanide, a drug used in the veterinary setting to treat fluke infections in ruminants, has in vitro activity in combination with colistin against colistin resistant Gram-negative bacilli (GNB) infections [13].

Pentamidine isethionate, an antiprotozoal drug commonly used in the treatment of trypanosomiasis, leishmaniosis, and fungal infections [14] was recently studied as antimicrobial agent. Stokes et al. reported that pentamidine showed synergism in combination with antibiotics against a wide range of GNB in vitro, and in a systemic murine infection in mice caused by *A. baumannii* [15]. Likewise, pentamidine alone and in combination with antimicrobials showed a robust in vitro activity against carbapenemase-producing and/or colistin-resistant Enterobacteriaceae [14]. The synergy of pentamidine in combination with antibiotics may be explained because of the pentamidine mechanism of action involves outer-membrane disruption in Gram-negative bacterium [15] so it could act as an adjuvant agent capable of sensitizing Gram-negative bacteria to antibiotics with intracellular mechanisms of resistance. However, pentamidine alone is not active against Gram-negative bacilli [15]. Another study analyzed the mechanisms of pentamidine resistance in *A. baumannii*, finding that the type of carbon source and the availability of iron affect the pentamidine activity against *A. baumannii* [16].

Considering the worldwide MDR of *P. aeruginosa* and the in vitro and in vivo synergistic effect of pentamidine combined with antibiotics against other GNB, the aim of this study was to evaluate the in vitro activity of pentamidine alone and in combination with different antimicrobials to know if it could be used as an adjuvant to antibiotics commonly used to treat MDR *P. aeruginosa* infections.

## 2. Results

### 2.1. MIC/MBC and Heteroresistance

The MIC/MBC results of each antimicrobial tested for the different clinical strains are shown in Table 1. The range of MIC for the antibiotics was for amikacin 1–32 mg/L, for gentamicin 0.13–16 mg/L, for tobramycin 0.13–32 mg/L, for imipenem 8–64 mg/L, for meropenem and aztreonam 2–32 mg/L, for ceftazidime 1–512 mg/L, for ciprofloxacin 0.5–16 mg/L, for levofloxacin 1–64 mg/L, for colistin <0.06–4 mg/L, and for pentamidine 400–1600 mg/L. The clinical strains Pa194, Pa215, Pa223, and Pa249 were MDR [17], and the rest were resistant to two different families of antibiotics.

Heteroresistance was observed only with ciprofloxacin for the Pa29 isolate, in which the MIC change from 0.5 mg/L to 4 mg/L after 48 h of incubation at 37 °C.

### 2.2. Time–Kill Curves

The bactericidal activity of the drugs alone is shown in Table 2, indicating the hours of incubation (parentheses) with a reduction of ≥3 log_10_ CFU/mL. Pentamidine alone at concentration equivalent to 1×MIC showed bactericidal effect against six out of the seven strains. As detailed in the table, pentamidine at 1×MIC shows an early bactericidal activity in 4 of the 6 strains from 2 hours of incubation, and with duration of 24 h in two of the strains. As expected, due to its in vitro activity, the bactericidal activity of antibiotics tested was found only against four strains, and heterogeneous: tobramycin, meropenem, and levofloxacin against Pa194, levofloxacin against Pa206, amikacin and colistin against Pa215, and aztreonam and levofloxacin against Pa249. None of the antibiotics nor pentamidine showed bactericidal activity against the Pa223 strain.

The synergistic and bactericidal activities of the combinations of pentamidine with the antibiotics are shown in Table 3, indicating the hours of incubation (parentheses) with the synergistic or bactericidal effect. In summary, the combinations of pentamidine with the antibiotics increase the antibiotics in vitro activity when tested alone, except for the combination with colistin. The combinations more synergistic were pentamidine with imipenem or meropenem (six and five strains, respectively), followed by pentamidine with ciprofloxacin or levofloxacin (four and three strains, respectively), pentamidine plus amikacin, gentamicin, or tobramycin (three, two, and one strains, respectively), and pentamidine with ceftazidime or aztreonam (two and one strains, respectively). The synergy occurred at 2 h in three cases (imipenem, ciprofloxacin, and levofloxacin) against Pa249 and in one case (ciprofloxacin) against Pa215. In the case of the Pa223 isolate, pentamidine in combination with five of the antibiotics tested (amikacin, meropenem, aztreonam, ceftazidime, and ciprofloxacin) was synergistic at 24 h.

**Figure 1:**
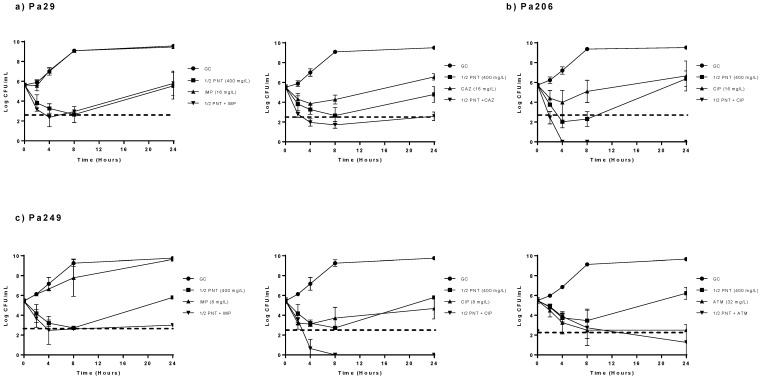
Time-kill curves for pentamidine at 1/2×MIC in combination with antibiotics against the clinical strains Pa29, Pa206 and Pa249. (**a**) Pa29, (**b**) Pa206 and (**c**) Pa249; GC: growth control, filled circles; pentamidine (PNT, 1/2xMIC), filled squares; antibiotics alone (IMP: imipenem, CAX: ceftazidime, CIP: ciprofloxacin; ATM: aztreonam, 1xMIC), filled triangles; PNT (1/2xMIC) + antibiotics (1xMIC), filled inverted triangles. Dotted line: indicates when and how long a compound alone or a combination has bactericidal activity.

The activity of pentamidine at sub-MIC concentration (1/2×MIC) in combination with the selected antibiotics is detailed in Figure 1. These results showed that pentamidine at sub-MIC (1/2×MIC) concentration improves the activity of the selected antibiotics when tested alone against the three selected strains. The combination with imipenem showed bactericidal activity at 4 h of incubation against the Pa29 and Pa249 strains. In the same way, pentamidine (1/2×MIC) plus aztreonam showed bactericidal activity at 24 h against the Pa249 isolate. When combined with ciprofloxacin we found bactericidal activity against the Pa206 from 2 to 24 h, and against the Pa249 from 4 to 24 h; moreover, this combination presented synergistic activity for Pa 206 and Pa249 from 8 to 24 h and from 4 to 24 h, respectively. Finally, the combination with ceftazidime against the Pa29 presented bactericidal and synergistic activity, from 4 to 24 h and at 24 h, respectively.

## 3. Discussion

This study evaluate in vitro the use of pentamidine as adjuvant to antibiotics commonly used to treat MDR *P. aeruginosa* infections, finding synergistic effect as well as bactericidal activity against most of the strains, when combined pentamidine with most of the tested antibiotics, such as aminoglycosides, carbapenems, aztreonam, ceftazidime, and fluoroquinolones, but not with colistin. Additionally, pentamidine at a sub-inhibitory concentration potentiated the in vitro activity of antibiotics with no in vitro activity when studied alone against three selected *P. aeruginosa* strains.

Pentamidine alone showed a high MIC values’ range, from 400 to 1600 mg/L, against the seven *P. aeruginosa* clinical strains. Given that pentamidine is currently used as an antifungal agent [18], there are no breakpoints to define the antibacterial susceptibility. However, the MIC values found in this study are in accordance with those reported in previous studies. In this sense, Stokes et al. analyzed the in vitro activity of pentamidine and five pentamidine analogs against an *Escherichia coli* strain, with MIC values ranging from 100 to >200 mg/L [15]. Also, the in vitro studies of pentamidine in combination with antibiotics, against clinical carbapenemase-producing and/or colistin-resistant Enterobacteriaceae, showed a pentamidine MIC value from 200 to 800 mg/L [14,15,19].

When pentamidine was analyzed alone, in time-kill curves at concentrations equivalent to its MIC values, we found that it exhibits bactericidal activity against six of the seven tested strains. This data, is in accordance with the ones in which pentamidine alone at MIC concentrations showed bactericidal activity in vitro against carbapenemase-producing and/or colistin-resistant Enterobacteriaceae [16], and with other studies showing synergistic effect with pentamidine in combination with rifampicin against a wide number of antibiotic-resistant strains, including *A. baumannii* and *E. coli* [15]. Additionally, a recent study demonstrated that pentamidine can potentiate non-antibiotic drugs achieving synergism and a decreasing bacterial load against GNB [20].

In the work of Stokes et al. [15] they stated that the primary mechanism of action of pentamidine could involve the outer-membrane disruption in the specific context of a Gram-negative bacterium. Additionally, they found in an *E. coli* strain expressing a truncated lipopolysaccharide variant no enhanced activity of pentamidine when analyzed alone, fact that would not support the hypothesis of pentamidine activity on an intracellular target. Nevertheless, this finding was supported by experiments using only one strain of *E. coli*. In our study, we have analyzed, using seven strains of *P. aeruginosa*, if pentamidine enhances the activity of antibiotics to which the strains were resistant. In this sense, our data shows that pentamidine, at concentrations equivalent to its MIC, increased the activity of most antibiotics tested against the MDR clinical *P. aeruginosa* strains, regardless if their mechanism of action, intracellular or on the bacterial membrane. Therefore, we observed that this activity was enhanced in antibiotics with intracellular mechanisms of action, such as aminoglycosides (amikacin, gentamicin and tobramycin) and fluoroquinolones (ciprofloxacin and levofloxacin), but it did also inhibit antibiotics that act by inhibiting the synthesis of the cell wall-like carbapenems (imipenem and meropenem) or cephalosporins (ceftazidime). Furthermore, and regardless of the mechanism of action of carbapenems, pentamidine plus imipenem and meropenem were the combinations that showed synergistic activity against more strains, six and five out of the seven strains tested. These data suggest that further studies are necessary to elucidate the mechanisms of action of pentamidine in *P. aeruginosa*.

Moreover, our data shows that antibiotics that alone did not have in vitro activity when combined with a sub-inhibitory pentamidine concentration showed bactericidal and synergistic activity against the tested strains. The present results are in accordance to the ones showed by Cebrero-Cangueiro et al., in which synergism was observed with pentamidine plus antibiotics against carbapenemase-producing and/or colistin-resistant Enterobacteriaceae [16]. Stokes et al. showed a synergistic effect with pentamidine in combination with rifampicin against a wide number of antibiotic-resistant strains, including *A. baumannii* and *E. coli* [15].

In summary, our results show in vitro synergy of pentamidine in combination with several families of antibiotics against MDR *P. aeruginosa*, suggesting the possible role of pentamidine, as a repurposed drug, for antibacterial use against these difficult to treat MDR pathogens.

## 4. Materials and Methods

### 4.1. Bacterial Isolates

Seven MDR (according to the standard clinical microbiology methods) clinical isolates of *P. aeruginosa* collected from different samples, during the quasi-experimental ecological study CARBAPIRASOA (RAM-PIR-2016-01) were studied: Pa29 from blood; Pa194 from pharyngeal exudate; Pa206 from surgical wound; Pa215 from skin biopsy; Pa249 from venous catheter; and Pa223 and Pa302 from bronchial aspirates. The Pa194 isolate is an IMP-3 producer.

### 4.2. Drugs

Pentamidine isethionate and the nine antibiotics (amikacin, gentamicin, tobramycin, imipenem, meropenem, aztreonam, ceftazidime, levofloxacin and colistin) were used as standard laboratory powders from Sigma (Sigma-Aldrich, Madrid, Spain), except for ciprofloxacin, which was from Fluka (Fluka BioChemika, Buchs, Switzerland). All the powder drugs were resuspended according to laboratory recommendations for in vitro uses.

### 4.3. Antimicrobial Susceptibility Testing

To confirm the data from the standard clinical microbiology studies on susceptibility and resistance of the selected strains, the MICs of pentamidine and the nine antibiotics were determined by broth microdilution as recommended by the Clinical and Laboratory Standards Institute (CLSI) [21], using Mueller Hinton broth II (Becton Dickinson & Co., Sparks, MD, USA) and an inoculum of approximately 5 × 10^5^ CFU/mL. The MIC was evaluated after approximately 18–24 h of incubation at 37 °C and was defined as the minimal concentration of antibiotic that was able to inhibit macroscopic bacterial growth. The MIC results were interpreted according to the European Committee on Antimicrobial Susceptibility Testing (EUCAST) [22] breakpoints for all antibiotics, except for gentamicin, which was interpreted according to the CLSI recommendations [21], and pentamidine, which has no defined susceptibility breakpoints. MIC breakpoints used to designate susceptibility and resistance, respectively, were: amikacin ≤16 mg/L and >16 mg/L; gentamycin ≤4 mg/L and ≥16 mg/L; tobramycin ≤2 mg/L and >2 mg/L; imipenem ≤0.001 mg/L and >4 mg/L; meropenem ≤2 mg/L and >8 mg/L; aztreonam ≤0.001 mg/L and >16 mg/L; ceftazidime ≤0.001 mg/L and >8 mg/L; ciprofloxacin ≤ 0.001 mg/L and >0.50 mg/L; levofloxacin ≤0.001 mg/L and >1 mg/L; and colistin ≤2 mg/L and >2 mg/L. *P. aeruginosa* ATCC 27853 was used as quality control strain.

Minimum bactericidal concentrations (MBCs) were determined by subculturing 100-μL aliquots from inoculated tubes containing antimicrobial concentrations higher than or equal to the MIC of each agent on to antibiotic-free Mueller–Hinton agar. Plates were incubated at 37 °C for 24 h, and colonies were counted. The MBC was defined as the concentration that killed ≥99.9% of the initial inoculum [14].

The heteroresistance assay was also carried out by reading the MIC after 48 h of incubation. Bacterial strains were defined as heteroresistant when they were able to grow at least two dilutions above the previously determined MIC value [14]. All antimicrobial susceptibility assays were performed in triplicate in different days to confirm the reproducibility.

### 4.4. Bactericidal Activity and Synergy Studies

Bactericidal and synergistic activities of pentamidine, the antibiotics, and pentamidine plus each antibiotic were performed using bacteria in the log-phase according to standard recommendations [22], by time–kill curves. In vitro time-kill curves were performed only for the strains that were resistant to the different antibiotics. We used 20 mL of MHB medium, an inoculum of approximately 5 × 10^5^ CFU/mL, and prefixed concentrations of 1×MIC for the antibiotics and pentamidine. Bacterial growth was quantified after 0, 2, 4, 8, and 24 h of incubation at 37 °C by plating 10-fold dilutions on sheep blood agar. To avoid carryover antimicrobial agent interference, the sample was placed on the plate in a single streak down the center, allowed to absorb into the agar until the plate surface appeared dry, and the inoculum was then spread over the plate. The limit of detection was 10 colony-forming unit (CFU)/mL, corresponding to 1 log_10_ CFU/mL [16].

Bactericidal effect was defined by a decrease of ≥3 log_10_ CFU/mL from the initial inoculum; a bacteriostatic effect was established as no change with respect to the initial bacterial concentration during the 24 h, and synergistic effect was defined as a decrease of ≥2 log_10_ CFU/mL for combined drugs compared with the most active single agent [23]. The experiments were performed three times on separate occasions.

Moreover, and after the results obtained with the time-kill curves studies at concentrations equivalent to 1×MIC of the drugs, we selected three strains (Pa29, Pa206, and Pa249) to evaluate if at sub-MIC pentamidine concentration (1/2×MIC) there was still in vitro synergy when combined with selected antibiotics which did not show in vitro activity when tested alone.

## 5. Conclusions

In conclusion, these results provide new insights regarding the repurpose of pentamidine as adjuvant to potentiate the activity of antibiotics commonly used to treat MDR *P. aeruginosa* infections, suggesting this combination as a new alternative in the treatment of infections by this pathogen. Further steps, including pharmacokinetic and pharmacodynamic studies and in vivo efficacy evaluation in experimental models of infection, including the dosage and safety, are required to confirm the usefulness of pentamidine in the treatment of infection by MDR *P. aeruginosa*.

## Figures and Tables

**Table 1:** MIC/MBC of the different antimicrobials for the seven MDR *Pseudomonas aeruginosa* clinical strains.

Clinical Strains	MIC/MBC (mg/L)										
	PNT	AMK	GEN	TOB	IMP	MEM	ATM	CAZ	CIP	LFX	COL
Pa29	800	1/2	1/1	0.25/0.25	16/16	32/32	16/64	16/32	0.50/4	1/1	0.13/1
Pa194	400	16/16	16/>32	32/>32	64/64	32/>64	2/8	512/512	1/1	32/64	0.50/1
Pa206	800	1/2	0.13/0.13	0.50/1	8/8	16/16	16/16	2/4	16/32	64/>64	0.13/0.13
Pa215	1600	32/32	8/16	2/2	8/8	32/32	16/32	32/64	4/8	16/64	4/8
Pa223	400	32/32	1/1	1/1	32/32	16/16	16/64	32/128	1/2	2/16	<0.06/<0.06
Pa249	800	2/2	0.13/0.13	0.13/0.25	8/8	16/32	32/32	8/8	8/16	32/64	0.25/0.5
Pa302	400	2/4	1/2	0.25/0.5	8/8	2/4	8/32	1/4	0.5/1	2/8	2/8

PNT: pentamidine; AMK: amikacin; GEN: gentamicin; TOB: tobramycin; IMP: imipenem; MEM: meropenem; ATM: aztreonam; CAZ: ceftazidime; CIP: ciprofloxacin; LFX: levofloxacin; COL: colistin. Pentamidine breakpoints are not defined; AMK: Susceptible MIC ≤ 16 mg/L and resistant MIC > 16 mg/L; GEN Susceptible MIC ≤ 4 mg/L and resistant MIC ≥ 16 mg/L; TOB: Susceptible MIC ≤ 2 mg/L and resistant MIC > 2 mg/L; IMP: Susceptible MIC ≤ 0.001 mg/L and resistant MIC > 4 mg/L; MEM: Susceptible MIC ≤ 2 mg/L and resistant MIC > 8 mg/L; ATM: Susceptible MIC ≤ 0.001 mg/L and resistant MIC > 16 mg/L; CAZ: Susceptible MIC ≤ 0.001 mg/L and resistant MIC > 8 mg/L; CIP: Susceptible MIC ≤ 0.001 mg/L and resistant MIC > 0.50 mg/L; LFX: Susceptible MIC ≤ 0.001 mg/L and resistant MIC > 1 mg/L; COL: Susceptible MIC ≤ 2 mg/L and resistant MIC > 2 mg/L.

**Table 2:** Bactericidal activity of antimicrobials alone against the seven *Pseudomonas aeruginosa* clinical strains.

Clinical Strain	PNT	AMK	GEN	TOB	IMP	MEM	ATM	CAZ	CIP	LFX	COL
Pa29	B (2–8)	-	-	-	NA	NA	-	NA	-	-	-
Pa194	B (2–8)	NA	NA	B (2–8)	NA	B (8)	-	NA	NA	B (2–24)	-
Pa206	B (2–24)	-	-	-	NA	NA	-	-	NA	B (8–24)	-
Pa215	B (2–4)	B (8)	NA	-	NA	NA	-	NA	NA	NA	B (2–8)
Pa223	NA	NA	-	-	NA	NA	-	NA	NA	NA	-
Pa249	B (8)	-	-	-	NA	NA	B (8–24)	-	NA	B (2–24)	-
Pa302	B (8–24)	-	-	-	NA	-	-	-	-	NA	-

PNT: pentamidine; AMK: amikacin; GEN: gentamicin; TOB: tobramycin; IMP, imipenem; MEM: meropenem; ATM: aztreonam; CAZ: ceftazidime; CIP: ciprofloxacin; LFX: levofloxacin; COL: Colistin. B: bactericidal; NA: no bactericidal activity; (): time frame in hours of the bactericidal in vitro activity; -: analysis not performed.

**Table 3:** Synergistic and bactericidal activities of pentamidine in combination with antibiotics against the seven *Pseudomonas aeruginosa* clinical strains.

Clinical Strain	PNT + AMK	PNT + GEN	PNT + TOB	PNT + IMP	PNT + MEM	PNT + ATM	PNT + CAZ	PNT + CIP	PNT + LFX	PNT + COL
Pa29	-	-	-	B (2–24) S (24)	B (2–24) S (24)	-	B (4–24)	-	-	-
Pa194	B (4–24) S (8–24)	B (2–8) S (8–24)	B (2–24) S (24)	B (2–24) S (24)	B (2–24) S (24)	-	B (2–24) S (24)	B (4–24) S (8–24)	B (2–24)	-
Pa206	-	-	-	B (2–24)	B (8–24) S (24)	-	-	B (2–24)	B (2–24)	-
Pa215	B (2–24) S (8–24)	B (2–24) S (8–24)	-	B (2–8) S (8)	B (2–24)	-	B (2–8)	B (2–24) S (2–24)	B (2–24) S (8–24)	B (2–8)
Pa223	B (24) S (24)	-	-	B (2–24) S (8–24)	B (2–24) S (24)	B (4–24) S (24)	B (4–24) S (24)	B (4–24) S (24)	B (2–24)	-
Pa249	-	-	-	B (2–24) S (2–24)	B (2–24) S (24)	B (8–24)	-	B (2–24) S (2–24)	B (2–24) S (2–24)	-
Pa302	-	-	-	B (4–24) S (8)	-	-	-	-	B (8–24) S (24)	-

PNT: pentamidine; AMK: amikacin; GEN: gentamicin; TOB: tobramycin; IMP, imipenem; MEM: meropenem; ATM: aztreonam; CAZ: ceftazidime; CIP: ciprofloxacin; LFX: levofloxacin; COL: Colistin. B: bactericidal; S, synergistic; (): time frame in hours of the in vitro activity found.

## References

[1] Botelho J., Grosso F., Peixe L. (2019). Antibiotic resistance in *Pseudomonas aeruginosa*—Mechanisms, epidemiology and evolution. Drug Resist. Updates Rev. Comment. Antimicrob. Anticancer Chemother..

[2] Khan F., Pham D.T.N., Oloketuyi S.F., Kim Y.M. (2020). Regulation and controlling the motility properties of Pseudomonas aeruginosa. Appl. Microbiol. Biotechnol..

[3] Tacconelli E., Carrara E., Savoldi A., Harbarth S., Mendelson M., Monnet D.L., Pulcini C., Kahlmeter G., Kluytmans J., Carmeli Y. (2018). Discovery, research, and development of new antibiotics: The WHO priority list of antibiotic-resistant bacteria and tuberculosis. Lancet Infect. Dis..

[4] Yoshimura K., Yano I., Yamamoto T., Kawanishi M., Isomoto Y., Yonezawa A., Kondo T., Takaori-Kondo A., Matsubara K. (2018). Population pharmacokinetics and pharmacodynamics of mycophenolic acid using the prospective data in patients undergoing hematopoietic stem cell transplantation. Bone Marrow Transplant..

[5] Surveillance Report (2018). Surveillance of Antimicrobial Resistance in Europe. https://www.ecdc.europa.eu/sites/default/files/documents/surveillance-antimicrobial-resistance-Europe-2018.pdf.

[6] Khodare A., Kale P., Pindi G., Joy L., Khillan V. (2020). Incidence, Microbiological Profile, and Impact of Preventive Measures on Central Line-associated Bloodstream Infection in Liver Care Intensive Care Unit. Indian J. Crit. Care Med. Peer-Rev. Off. Publ. Indian Soc. Crit. Care Med..

[7] Tabak Y.P., Merchant S., Ye G., Vankeepuram L., Gupta V., Kurtz S.G., Puzniak L.A. (2019). Incremental clinical and economic burden of suspected respiratory infections due to multi-drug-resistant Pseudomonas aeruginosa in the United States. J. Hosp. Infect..

[8] Morris S., Cerceo E. (2020). Trends, Epidemiology, and Management of Multi-Drug Resistant Gram-Negative Bacterial Infections in the Hospitalized Setting. Antibiotics.

[9] Pang Z., Raudonis R., Glick B.R., Lin T.J., Cheng Z. (2019). Antibiotic resistance in Pseudomonas aeruginosa: Mechanisms and alternative therapeutic strategies. Biotechnol. Adv..

[10] Miro-Canturri A., Ayerbe-Algaba R., Smani Y. (2019). Drug Repurposing for the Treatment of Bacterial and Fungal Infections. Front. Microbiol..

[11] Brown D. (2015). Antibiotic resistance breakers: Can repurposed drugs fill the antibiotic discovery void?. Nat. Rev. Drug Discov..

[12] Rampioni G., Visca P., Leoni L., Imperi F. (2017). Drug repurposing for antivirulence therapy against opportunistic bacterial pathogens. Emerg. Top. Life Sci..

[13] Ayerbe-Algaba R., Gil-Marques M.L., Miro-Canturri A., Parra-Millan R., Pachon-Ibanez M.E., Jimenez-Mejias M.E., Pachon J., Smani Y. (2019). The anthelmintic oxyclozanide restores the activity of colistin against colistin-resistant Gram-negative bacilli. Int. J. Antimicrob. Agents.

[14] Cebrero-Cangueiro T., Alvarez-Marin R., Labrador-Herrera G., Smani Y., Cordero-Matia E., Pachon J., Pachon-Ibanez M.E. (2018). In vitro Activity of Pentamidine Alone and in Combination With Aminoglycosides, Tigecycline, Rifampicin, and Doripenem Against Clinical Strains of Carbapenemase-Producing and/or Colistin-Resistant Enterobacteriaceae. Front. Cell. Infect. Microbiol..

[15] Stokes J.M., MacNair C.R., Ilyas B., French S., Cote J.P., Bouwman C., Farha M.A., Sieron A.O., Whitfield C., Coombes B.K. (2017). Pentamidine sensitizes Gram-negative pathogens to antibiotics and overcomes acquired colistin resistance. Nat. Microbiol..

[16] Adams F.G., Stroeher U.H., Hassan K.A., Marri S., Brown M.H. (2018). Resistance to pentamidine is mediated by AdeAB, regulated by AdeRS, and influenced by growth conditions in *Acinetobacter baumannii* ATCC 17978. PLoS ONE.

[17] Magiorakos A.P., Srinivasan A., Carey R.B., Carmeli Y., Falagas M.E., Giske C.G., Harbarth S., Hindler J.F., Kahlmeter G., Olsson-Liljequist B. (2012). Multidrug-resistant, extensively drug-resistant and pandrug-resistant bacteria: An international expert proposal for interim standard definitions for acquired resistance. Clin Microbiol. Infect..

[18] 2019 First Generic Drug Approvals. https://www.fda.gov/drugs/first-generic-drug-approvals/2019-first-generic-drug-approvals.

[19] Maciejewska D., Żabiński J., Kaźmierczak P., Wójciuk K., Kruszewski M., Kruszewska H. (2014). In vitro screening of pentamidine analogs against bacterial and fungal strains. Bioorg. Med. Chem. Lett..

[20] Wu C., Xia L., Huang W., Xu Y., Gu Y., Liu C., Ji L., Li W., Wu Y., Zhou K. (2020). Pentamidine sensitizes FDA-approved non-antibiotics for the inhibition of multidrug-resistant Gram-negative pathogens. Eur. J. Clin. Microbiol. Infect. Dis..

[21] CLSI (2019). Performance Standards for Antimicrobial Susceptibility Testing.

[22] EUCAST Breakpoint Tables for Interpretation of MICs and Zone Diameters Version 9.0. https://www.eucast.org/fileadmin/src/media/PDFs/EUCAST_files/Breakpoint_tables/v_9.0_Breakpoint_Tables.pdf.

[23] Pachón-Ibáñez M.E., Labrador-Herrera G., Cebrero-Cangueiro T., Díaz C., Smani Y., Del Palacio J.P., Rodríguez-Baño J., Pascual A., Pachón J., Conejo M.C. (2018). Efficacy of Colistin and Its Combination With Rifampin. Front. Microbiol..

